# When atrioventricular block paves the way to a more severe diagnosis: a case report

**DOI:** 10.1093/ehjcr/ytag152

**Published:** 2026-03-09

**Authors:** Ecaterina Argint, Dorina Cheibaș, Viorica Ochișor, Valeriu Revenco, Mihaela Agapii

**Affiliations:** Department of Cardiology, Nicolae Testemițanu State University of Medicine and Pharmacy, 165 Ștefan cel Mare și Sfânt Boulevard, Chișinău, Republic of Moldova; Republican Clinical Hospital ‘Timofei Moșneaga’, 29 Nicolae Testemițanu Street, Chișinău, Republic of Moldova; Department of Cardiology, Nicolae Testemițanu State University of Medicine and Pharmacy, 165 Ștefan cel Mare și Sfânt Boulevard, Chișinău, Republic of Moldova; Department of Cardiology, Nicolae Testemițanu State University of Medicine and Pharmacy, 165 Ștefan cel Mare și Sfânt Boulevard, Chișinău, Republic of Moldova; Department of Cardiology, Nicolae Testemițanu State University of Medicine and Pharmacy, 165 Ștefan cel Mare și Sfânt Boulevard, Chișinău, Republic of Moldova

**Keywords:** Systemic sclerosis, Complete atrioventricular block, Pulmonary arterial hypertension, Pulmonary fibrosis, Gastrointestinal involvement, Case report

## Abstract

**Background:**

Systemic sclerosis (SSc) is an autoimmune connective tissue disease characterized by widespread fibrosis of skin and internal organs. Cardiac involvement is common but often subclinical, and advanced conduction abnormalities such as complete atrioventricular block are rarely the initial manifestation.

**Case summary:**

A 42-year-old man presented with fatigue and episodes of syncope. Electrocardiography confirmed complete AV block. Physical examination revealed diffuse skin tightening and Raynaud’s phenomenon. Immunological testing detected high-titre antinuclear antibodies and anti-topoisomerase I, confirming diffuse cutaneous SSc. Further evaluation disclosed multiorgan involvement: high-resolution CT showed pulmonary fibrosis and right heart catheterization confirmed severe precapillary pulmonary hypertension. The patient underwent permanent dual-chamber pacemaker implantation and was started on immunosuppressive therapy and pulmonary vasodilators. On follow-up, his bradycardia resolved and exercise tolerance improved, indicating partial clinical improvement.

**Discussion:**

This case highlights an atypical cardiac onset of diffuse SSc with high-grade conduction disease. It underscores the importance of considering systemic autoimmune disorders in patients with unexplained high-degree AV block, especially in the presence of characteristic skin findings. Comprehensive assessment revealed concurrent pulmonary and gastrointestinal involvement, reflecting the multisystem nature of SSc. Early recognition allowed a coordinated multidisciplinary treatment strategy, including device implantation and disease-modifying therapy, which ultimately stabilized the patient’s condition. This case emphasizes the need for early diagnosis and integrated management of cardiac, pulmonary, and gastrointestinal complications in SSc. Although SSc is generally progressive, prompt recognition and treatment of organ involvement may significantly improve patient outcomes and quality of life.

Learning points

- Clinical context is crucial when interpreting ECG abnormalities to guide appropriate diagnostic pathways.
- Multidisciplinary management including pacemaker implantation and targeted SSc therapy can improve symptoms; early recognition and treatment of complications are essential.

## Introduction

Systemic sclerosis (scleroderma; SSc) is a rare autoimmune disease characterized by progressive fibrosis of the skin and internal organs, vasculopathy, and abnormal activation of the immune system. The disease is classified into two main clinical forms: limited and diffuse, depending on the extent of cutaneous involvement and the risk of visceral organ damage.^1^

The diffuse form is marked by rapid progression, early involvement of internal organs (lungs, heart, kidneys, gastrointestinal tract), and a higher mortality rate. Cardiac involvement, although often underdiagnosed, is a major adverse prognostic factor and may include conduction disturbances, ventricular dysfunction, and pericardial effusions. Complete atrioventricular block (CAVB) is a rare initial manifestation and requires careful differential diagnosis, particularly in young patients with no known medical history.^2,3^

## Summary figure

**Figure:**
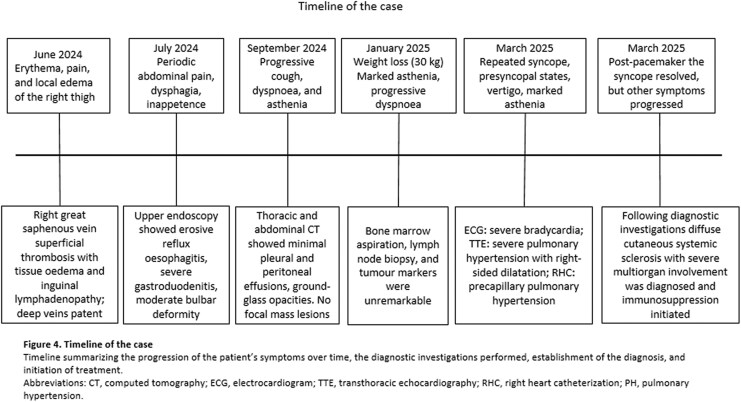


## Case presentation

A 42-year-old male with no significant past medical or family history presented with progressive deterioration of his general condition over 10 months, including approximately 30 kg weight loss, severe asthenia, dry cough, and exertional dyspnoea unresponsive to symptomatic treatment. One week prior to hospital admission (19 March 2025, IMSP Institute of Cardiology, tertiary-level hospital), he experienced sudden vertigo and recurrent syncopal episodes.

**Figure 1:**
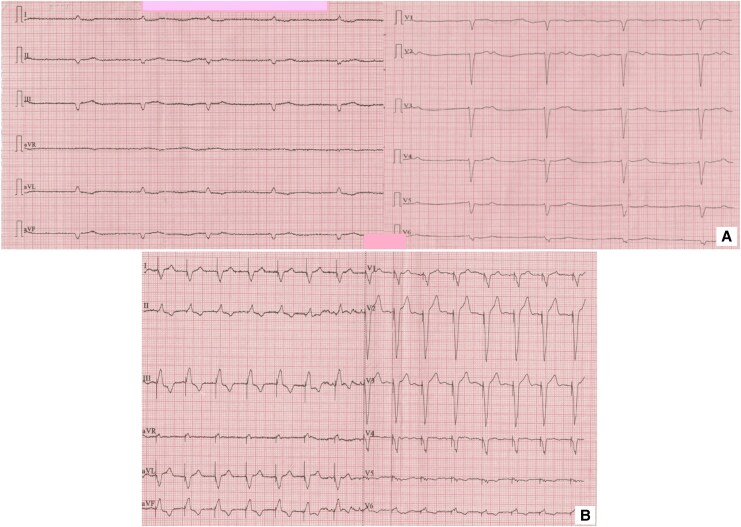
Electrocardiogram of the patient. (*A*) On admission, third-degree atrioventricular block with a heart rate of 32 bpm. (*B*) Electrocardiogram after pacemaker implantation.

The electrocardiogram (ECG) revealed marked bradycardia (32 bpm) with regular QRS complexes and a slow ventricular rate. Atrial activity was poorly discernible and showed no consistent temporal relationship with ventricular depolarization, raising suspicion of atrioventricular dissociation. While the ECG pattern suggested a severe atrioventricular conduction abnormality, possibly caused by CAVB, the differential diagnosis included sinus arrest with a junctional escape rhythm, first-degree atrioventricular block with a markedly prolonged PR interval, isorhythmic atrioventricular dissociation, and retrograde atrial activation. The absence of underlying sinus tachycardia may indicate associated sinus node dysfunction (*Figure 1A*). In view of the patient’s haemodynamically significant bradycardia, urgent dual-chamber pacemaker implantation was performed (*Figure 1B*).

Given the patient’s young age and lack of obvious causes for CAVB, an extended diagnostic workup was initiated.

**Figure 2:**
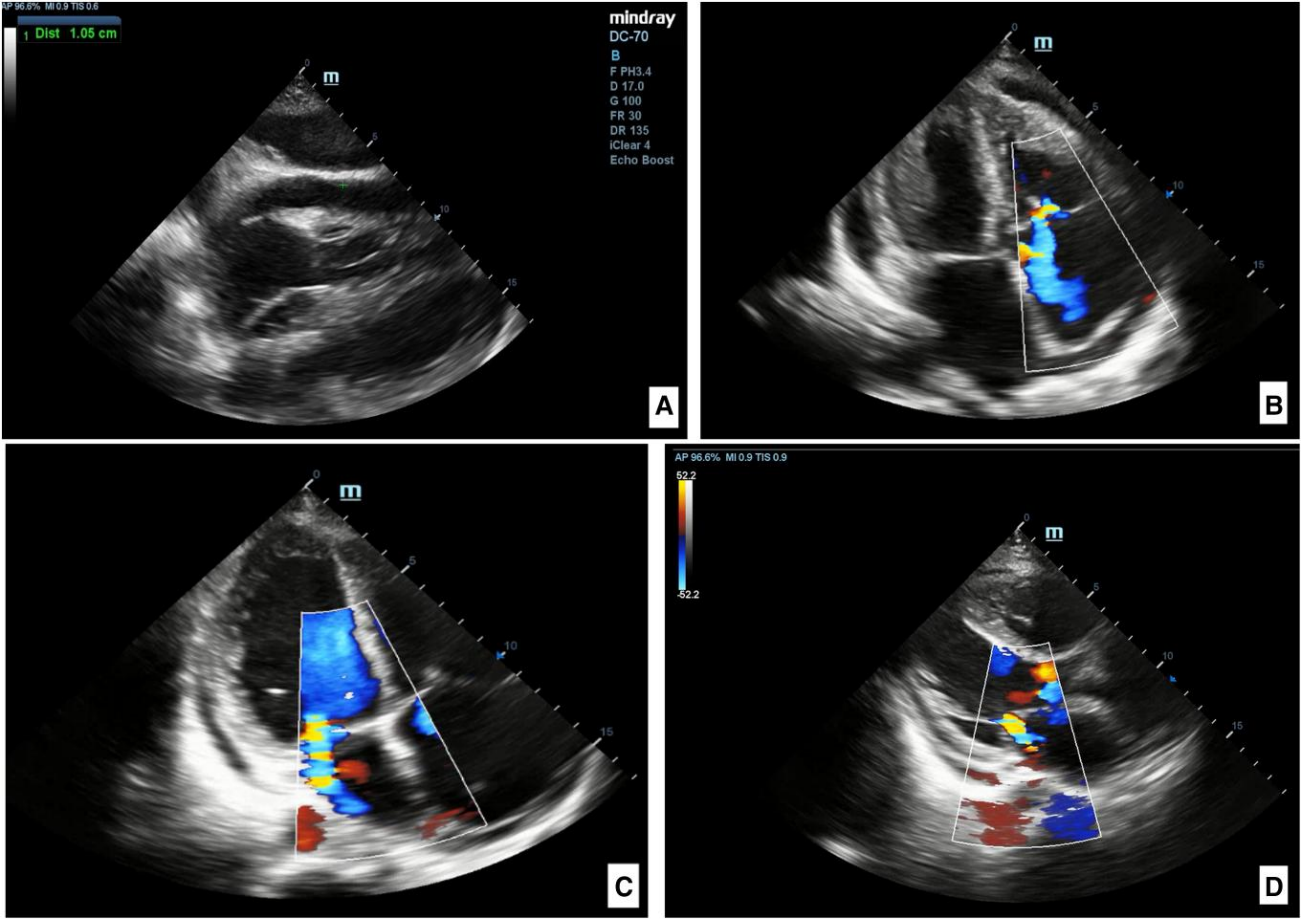
Echocardiography: (*A*) pericardial effusion, (*B*) moderate-to-severe tricuspid regurgitation, and (*C*, *D*) moderate mitral regurgitation.

Physical examination revealed features suggestive of SSc: sclerodactyly, indurated skin oedema, diffuse hyperpigmentation, and Raynaud’s phenomenon. The patient also reported progressive oropharyngeal dysphagia, dysphonia, heartburn, and diffuse myalgia. Echocardiography showed left ventricular ejection fraction 47%, moderate mitral and moderate-to-severe tricuspid regurgitation, severe pulmonary hypertension, significant right ventricular (mid-RV diameter 39 mm) and right atrial (RA 54 mm) dilatation, and moderate pericardial effusion. Pericardial fluid thickness measured 5 mm at the left ventricular posterior septum, 8 mm at the right ventricular posterior septum, 15 mm at the RA free wall, and 4 mm at the left ventricular posterolateral wall (*Figure 2*).

**Figure 3:**
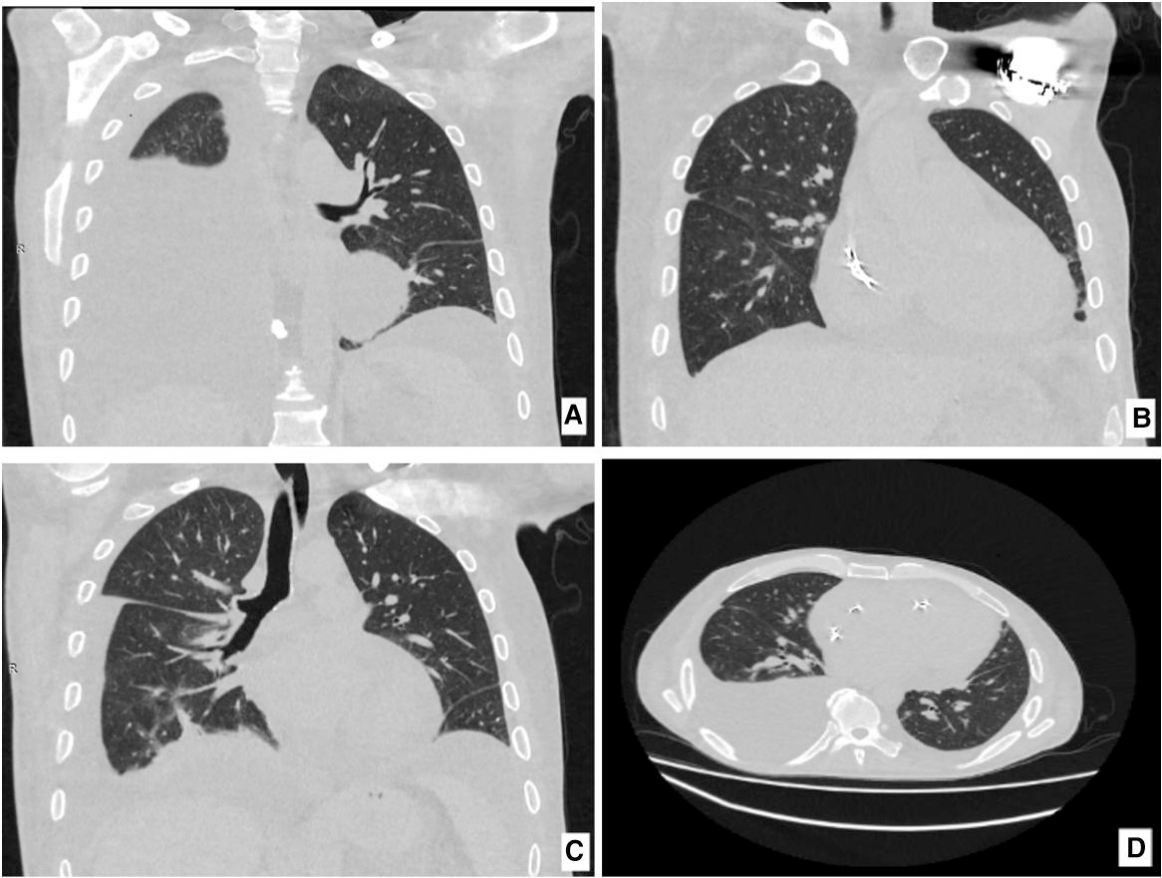
Pulmonary computed tomography. Pneumofibrotic changes and linear atelectasis in the bilateral basal pulmonary regions, free fluid in the bilateral pleural cavities (right: 1050 mL; left: minimal amount), free fluid in the pericardial cavity, and free fluid in the abdominal cavity.

Right heart catheterization demonstrated an elevated mean pulmonary arterial pressure of 36.4 mmHg, an increased right atrial pressure of 10.6 mmHg, a normal pulmonary arterial wedge pressure of 13.4 mmHg, and a markedly increased pulmonary vascular resistance of 6.8 Wood units. Cardiac output was preserved at 5.3 L/min, with a borderline normal cardiac index of 2.5 L/min/m^2^. CT pulmonary angiography revealed bilateral basal pulmonary fibrosis with linear atelectasis, right pleural effusion (∼1050 mL) with minimal left-sided fluid, moderate pericardial effusion, and a small amount of intra-abdominal fluid (*Figure 3*).

Laboratory tests showed markedly elevated inflammatory markers (CRP 96 mg/L [ref 0–5], ESR 42 mm/h [ref 2–10]), increased muscle enzymes (CK 396 U/L [ref 24–195], CK-MB 43 U/L [ref 0–24]), NT-proBNP 3753 pg/mL [ref 0–300], and persistently elevated troponin (2.00–2.32 ng/mL [ref 0–0.10]). Immunologic testing revealed anti-Scl-70 (anti-topoisomerase I) antibodies (45 U/mL [ref <15]) and high-titre ANA, with negative anti-centromere and anti-RNP antibodies. Tumour markers (CEA, AFP, CA 19-9, CA 125, PSA, hCG) were normal. Based on the 2013 ACR/EULAR criteria,^4^ diffuse cutaneous SSc with severe multiorgan involvement was diagnosed.

After rhythm stabilization, the patient was transferred to the tertiary Rheumatology Department, where immunosuppressive therapy was initiated: cyclophosphamide 0.5 g/m^2^ i.v., low-dose prednisone 0.3 mg/kg/day, vasodilators (nifedipine retard 20 mg—¼ tablet twice daily), proton pump inhibitor (pantoprazole 40 mg twice daily), loop diuretics (furosemide 20 mg i.v. daily), and prophylactic anticoagulation (enoxaparin 0.4 mL b.i.d.). After 3 months, a slight clinical improvement was observed, with reduced pleural effusion (∼100 mL), regression of pericardial effusion, and improved exercise tolerance. Five months after diagnosis, the patient died at home from multiorgan failure secondary to irreversible cardiopulmonary damage.

## Discussion

Diffuse cutaneous SSc is characterized by aggressive progression and multiorgan involvement. Cardiac involvement is a major prognostic determinant, accounting for approximately 25% of SSc-related deaths.^2^ Pulmonary manifestations, particularly interstitial lung disease (ILD) and pulmonary arterial hypertension (PAH), represent the leading causes of mortality, responsible for over 65% of SSc-related deaths in European cohorts (ILD 34.4%, PAH 31.2%).^5,6^ In this case, advanced pulmonary fibrosis on computed tomography and severe PAH indicated a poor prognosis, in line with published data.

Cardiac involvement in SSc includes arrhythmias and conduction disturbances; however, CAVB is rare, occurring in <2% of cases.^2^ Cardiac manifestations may remain clinically silent, but when CAVB occurs, permanent pacemaker implantation is usually required.^2^

Pacemaker implantation is recommended in SSc-related CAVB, including in asymptomatic patients, to prevent severe complications. In our case, despite dual-chamber pacemaker implantation, the outcome was unfavourable, highlighting that pacing does not modify prognosis in severe diffuse SSc, where PAH, pulmonary fibrosis, and gastrointestinal involvement are decisive. Cardiac involvement increases mortality risk approximately fivefold, with 5-year survival <30% in primary myocardial disease, and the need for pacemakers or ICDs is about twice that of the general population.^6^

Pulmonary involvement affects ∼80% of patients with SSc and is the leading cause of death and disability, while gastrointestinal involvement occurs in up to 90% and is a major source of morbidity.^7^ In our patient, interstitial pulmonary fibrosis was confirmed by computed tomography and gastrointestinal involvement by endoscopy. Multisystem disease, particularly severe gastrointestinal involvement, increases clinical complexity and may accelerate disease progression through malnutrition and secondary complications.

Cardiac magnetic resonance (CMR) imaging is recommended in patients with unexplained atrioventricular conduction disease to assess myocardial fibrosis, inflammation, or infiltrative processes, with important diagnostic and prognostic implications. In the present case, CMR was considered clinically relevant and was recommended; however, its implementation was limited by local technical availability.

Therapeutic management in SSc is organ-specific. Immunosuppressive therapy, including cyclophosphamide as used in our patient, is established for diffuse SSc with progressive pulmonary involvement.^3,4^ The 2023 EULAR recommendations support antifibrotic (nintedanib) and immunomodulatory therapies (mycophenolate mofetil, rituximab, tocilizumab) in severe pulmonary or cutaneous fibrosis.^3^ In SSc-associated PAH, targeted vasodilator therapies are indicated, but efficacy is limited in advanced disease, with 5-year survival rates below 25%.^6^

Registry and multicentre data show poor prognosis in SSc with delayed diagnosis. The SUISSE-EUSTAR cohort highlights the importance of early referral to specialized centres before irreversible organ damage occurs.^8^ Despite earlier diagnosis, disease severity and survival in Swiss patients are comparable to the European average. Overall survival is ∼97% at 1 year and 87% at 5 years,^5^ while male sex, cardiac involvement, reduced diffusing capacity for carbon monoxide, and systemic inflammation are associated with worse outcomes.

## Conclusion

Diffuse cutaneous systemic sclerosis is a complex autoimmune disease with high fatal potential in cases of extensive visceral involvement. Presentation with CAVB at onset may delay diagnosis, underscoring the need for a comprehensive, multisystem evaluation. Early therapeutic intervention—including aggressive immunotherapy, appropriate ventilatory/vascular support, and close follow-up—is essential to improve clinical outcomes in such severe cases.

## Data Availability

The data underlying this article are not publicly available due to ethical and privacy considerations, as they contain information that could compromise the confidentiality of the patient.

## Lead author biography

Dr Ecaterina Argint, MD, Associate Professor, is a faculty member at the Department of Cardiology of Nicolae Testemitanu State University of Medicine and Pharmacy and a cardiologist at the Institute of Cardiology, Chișinău, Republic of Moldova. She is actively involved in the daily training of numerous cardiology specialists, residents, and physicians from other disciplines. Dr Argint has advanced expertise in echocardiography and provides professional instruction and supervision in this field. Her clinical and academic activity is dedicated to improving patient care and advancing cardiovascular education through continuous teaching and mentorship.

**Consent:** Written informed consent was obtained from the patient for publication of this case report and any accompanying images. The authors confirm that this work complies with COPE guidelines.
